# Static T2w MRU in Noncalcular Urinary Obstruction: Comparison of Its Two Techniques

**DOI:** 10.1100/tsw.2008.66

**Published:** 2008-04-20

**Authors:** Mohamed Abou El-Ghar, Ahmed Shokeir, Huda Rafaie, Tarek El-Diasty

**Affiliations:** ^1^ Radiology Department, Urology and Nephrology Center, Mansoura University, Mansoura, Egypt; ^2^ Urology Department, Urology and Nephrology Center, Mansoura University, Mansoura, Egypt

**Keywords:** MRU, obstruction, static, single shot, multisection

## Abstract

The purpose of this study was to compare the diagnostic accuracy of T2-weighted (T2w) MR urography (MRU) techniques — the standard MRU using fast spin echo (FSE) and postprocessing maximum intensity projection (MIP) and the single-shot MRU — in the diagnosis of ureteric obstruction in patients with noncalcular urinary obstruction. The study included 150 patients admitted to our center between January 2005 and December 2006. There were 203 renal units with noncalcular obstruction; 53 patients had bilateral obstruction. Patients with calcular obstruction were excluded. There were 85 males and 65 females with a mean age of 50 (range: 5–83) years. All patients were examined with static MRU using both single-shot (thick slab) and multisection MRU. Using single-shot MRU, we obtained images at the direct coronal and oblique coronal, as well as sagittal, planes for each renal unit. Postprocessing MIP for the standard coronal heavy T2 source images to obtain coronal and oblique images was done. Among the obstructed 203 units, the intrinsic causes were present in 157 units (151 were stricture and six were ureteric tumors), while the extrinsic causes were present in 46 units (35 bladder tumor, four ureterocele, five retroperitoneal fibrosis, one prostatic tumor, and one local pelvic recurrence after radical cystectomy for bladder cancer). The overall accuracy of single-shot MRU was 89% and was 93% for the multisection MRU in cases of intrinsic ureteric obstruction, while in cases of extrinsic obstruction, it was 20% for single-shot MRU and 96% for multisection MRU. T2w static MRU is a very useful technique in diagnosing noncalcular ureteric obstruction. Multisection MRU has a high diagnostic accuracy and reliability over that of the single-shot technique. The single-section technique is very rapid and useful in diagnosing ureteric stricture so it could be used as a localizer, while multisection images with postprocessing MIP is mandatory, especially in cases of suspected ureteric tumors or extraureteric causes.

## INTRODUCTION

The concept of MR urography (MRU) came into existence at the end of 1980s. The static fluid MRU (sMRU), based on heavily $T_2$-weighted (T2w) images, visualizing static and slowly flowing fluids, was the first technique used in clinical practice[1,2].

MRU was described in cases of patients with obstructive uropathy. Heavily T2w sequences can visualize a dilated urinary tract without injection of contrast materials because MRU selectively depicts urine in the dilated renal collecting system and ureter[3,4,5,6,7].

In the clinical practice of T2w sMRU, single-slice projection imaging using rapid acquisition with relaxation enhancement (RARE) or half-Fourier acquisition single-shot turbo spin echo (HASTE) does not have the same discernment as the multislice technique. Multislice standard turbo spin echo (TSE) sequences are more time consuming, but have proved to be superior for the detection of small pathological details, which can be missed on 8-cm-thick projection single-shot MR urograms[5,8,9]. Single-shot MRU does not have the prerequisites of intravenous urography, such as ionizing radiation, injection of contrast media, and functioning kidney, but it has been hampered by limited spatial resolution[10,11,112,13]. A fast-echo pulse sequence adapted for single-shot use reduces the acquisition time for a full 250 × 256 matrix to 2.8 sec and permits selective projection of the collecting system in convenient breath holds. Whereas intravenous urography may last from 15 min to several hours, single-shot MRU is completed within a maximum of 5 min. Single-shot MRU allows full coverage of the collecting systems and a selective projective without postprocessing is achieved due to inherent signal loss of solid tissues[10,11]. Postprocessing required for a selective display in T2w pulse sequences with shorter echo trains bears a high risk of introducing artifacts and lengthens time to complete the examination[9].

The purpose of the present study was to compare the diagnostic accuracy of T2w MRU techniques, the standard MRU using FSE and postprocessing maximum intensity projection (MIP) with the single-shot MRU, in the diagnosis of ureteric obstruction in patients with noncalcular urinary obstruction.

## MATERIALS AND METHODS

The study included 150 patients admitted to our center between January 2005 and December 2006. There were 203 renal units with hydronephrotic changes due to noncalcular causes; 53 patients had bilateral hydronephrosis. Patients with calcular causes were excluded. There were 85 males and 65 females with a mean age of 50 (range: 5–83) years. Consent was received from all patients.

All patients were examined with static MRU using both single-shot (thick slab) and multisection MRU. Single-shot MRU parameters were: TR – 18,000 msec, TE – 700 msec, band width – 31–25, field of view (FOV) – 42, slice thickness – 100 mm. The multisection standard FSE MRU technique includes the coronal heavy T2 with the following parameter: TR – 10,000–16,000 msec, TE – 250 msec, number of excitation (NEX) – 2, FOV – 44, slice thickness – 4 mm, spacing – 1 mm; and complementary axial $T_2$ at the level of obstruction with the following parameters: TR – 9,000-15,000 msec, TE – 102 msec, band width – 83, NEX – 2, FOV – 40, slice thickness – 4 mm, spacing – 1 mm.

The study was done utilizing a superconductive MRI scanner 1.5T (Signs Horizon; General Electric Medical System, Milwaukee, WI).

Using single-shot MRU, we obtained images at the direct coronal and oblique coronal, as well as sagittal, planes for each renal unit; each acquisition was obtained with a breath hold of 3 sec. Postprocessing MIP was done at a special workstation (ADW 4.1, General Electric Medical System; Milwaukee, WI) for the standard coronal heavy T2 source images to obtain the coronal and oblique images.

The reference standards for the diagnosis were combined studies including antegrade and/or retrograde studies, as well as operative and endoscopic findings. No contrast material injection was used except in 20 patients where malignancy and retroperitoneal fibrosis were suspected.

## RESULTS

The study included 150 patients with 203 hydronephrotic changes due to noncalcular causes; 53 patients had bilateral hydronephrosis. The causes of hydronephrosis are shown in Table 1 and are classified into extrinsic and intrinsic causes. Intrinsic causes were present in 157 units: 151 were stricture and six were ureteric tumors. Extrinsic causes were present in 46 units: 35 bladder tumor, four ureterocele, five retroperitoneal fibrosis, one prostatic tumor, and one local pelvic recurrence after radical cystectomy for bladder cancer.

For intrinsic stricture, there is a smooth five tapering at MIP and single-shot MRU (fig. 1) with no mural irregularities or filling defect; stricture either at ureteropelvic junction (UPJ), ureter, or at ureterovesical junction (UVJ) levels. Filling defects for ureteric tumor could not be appreciated on both single-shot and MIP images because the hypointensity of the lesion could not be identified among the hyperintense fluids. Ureteric tumors were diagnosed at axial T2 MR images at the level of obstruction. Extrinsic causes of obstruction could be appreciated by ureteric displacement and concentric narrowing with gradual tapering of the ureter.

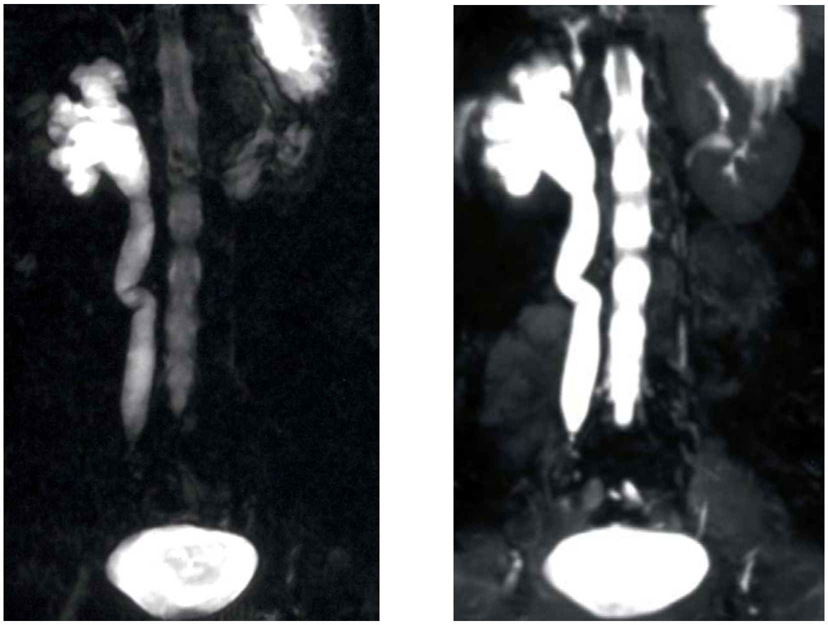

A B

**FIGURE 1.** A case of stricture at right iliac ureter. (A) Single shot. (B) MIP reformatted multisection MRU shows the stricture and its level adequately at both sequences.

The agreement between single-shot and standard MRU, including MIP, vs. gold standard in the diagnosis of both extrinsic and intrinsic causes of obstruction are given in Tables 2 and 3. The sites of obstruction were identified in all 203 units by single-shot and standard MRU.

Among the cases with stricture, there were 14 units with stricture at the UPJ, 100 units at the ureteric level, and 37 units at the UVJ. The strictures at the UPJ were identified in 14/14 using standard MRU and in 7/14 (50%) using single-shot MRU. While in cases of ureteric and UV stricture, single-shot and standard MRU could detect the cause in 127/137 (93%) with the same accuracy. Ureteric tumors diagnosed using standard MRU were 6/6, but none could be diagnosed using single-shot MRU (fig. 3).

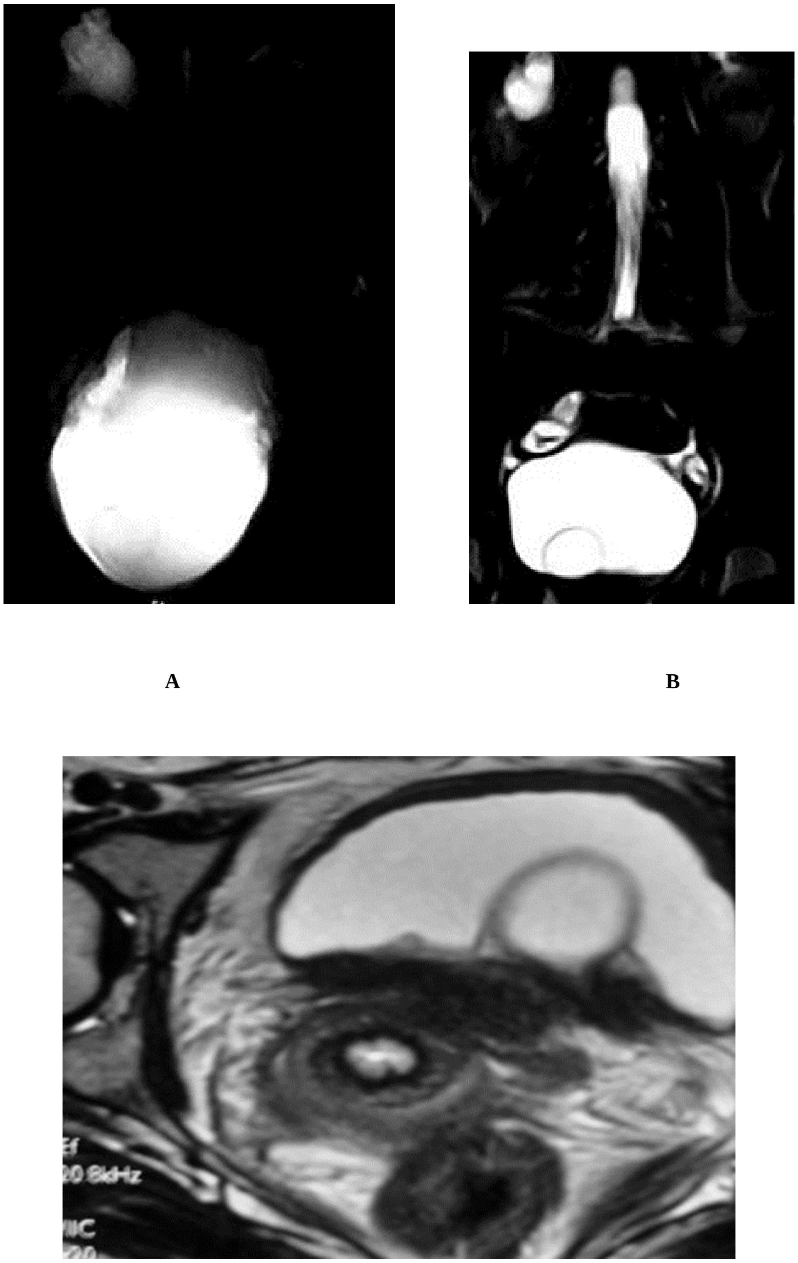

A B

C

**FIGURE 2.** A case of right intravesical ureterocele. (A) Single-shot MRU; the filling defect of the ureterocele could not be identified clearly. (B and C) Coronal source image of MRU and axial T2w at the level of urinary bladder; both show clearly the ureterocele inside the urinary bladder.

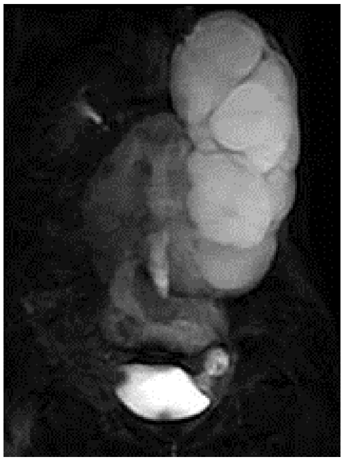

A

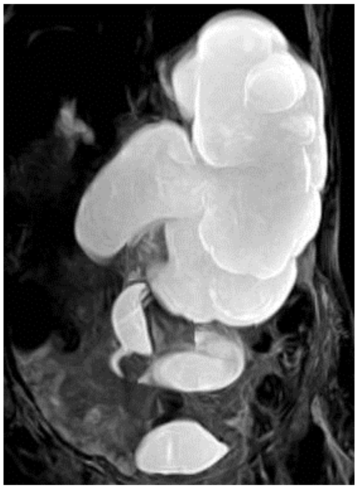

B

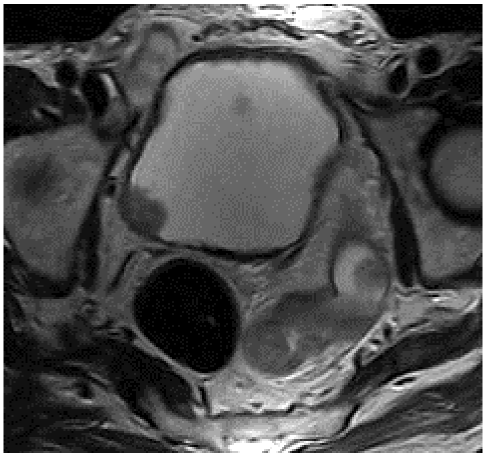

C

**FIGURE 3.** A case of multicentric intavesical and left ureteric transitional cell carcinoma. (A) Single-shot MRU shows ill-defined soft tissue lesions at urinary bladder. (B,C) Coronal reformatted MRU and axial T2w at the pelvis show the multicentric left ureteric and urinary bladder soft tissue masses.

**TABLE 1**
**The Final Diagnosis of 203 Renal Units with Noncalcular Urinary Obstruction**

| Obstruction | No. |
|---|---|
| Intrinsic | 157 |
| Stricture | 151 |
| Ureteric tumor | 6 |
| Extrinsic | 46 |
| Bladder carcinoma | 35 |
| Retroperitoneal fibrosis | 5 |
| Ureterocele | 4 |
| Prostatic carcinoma | 1 |
| Pelvic tumor recurrence | 1 |

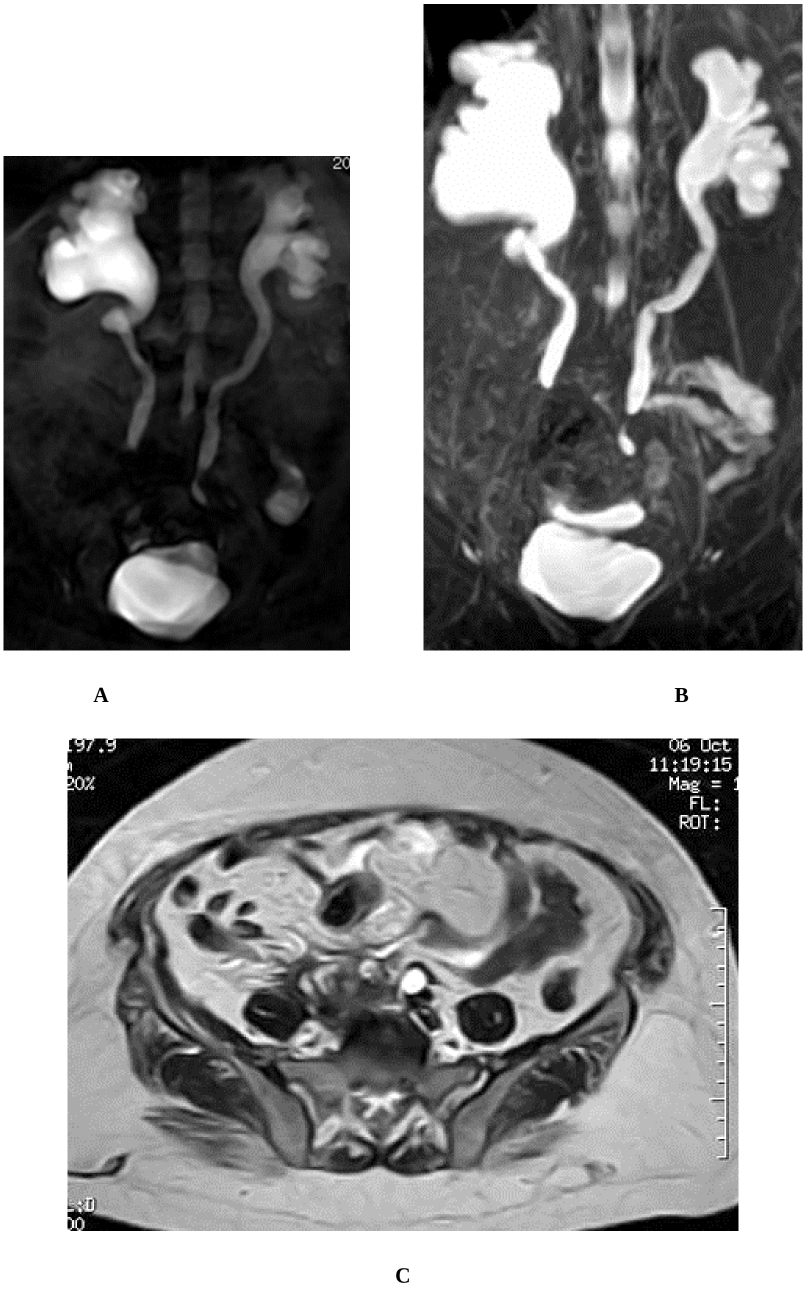

**FIGURE 4.** A case of bilateral ureteric obstruction secondary to retroperitoneal fibrosis. (A,B) Single-shot MRU and coronal reformatted MIP MRU show bilateral obstructed renal units down to the iliac ureters. (C) Axial T2w at the iliac region shows the retroperitoneal soft tissue mass involving both ureters at the pelvic region.

Among the extrinsic causes of urinary obstruction, standard MRU diagnosed 35/35 obstructions secondary to bladder tumor, while single-shot MRU diagnosed 7/35 (20%), 5/5 cases with retroperitoneal fibrosis were diagnosed using standard MRU (fig. 4), but none were diagnosed using single shot. One case of prostatic cancer and one of pelvic tumor local recurrence were not diagnosed using either of the techniques. In four cases with ureterocele (fig. 2), the standard MRU diagnosed 4/4, while single-shot MRU diagnosed 2/4 (50%).

The overall accuracy of single-shot MRU was 89% and was 93% for the multisection MRU in cases of intrinsic ureteric obstruction, while in cases of extrinsic obstruction, it was 20% for single-shot MRU and 96% for multisection MRU.

**TABLE 2**
**True Positive and Negative Values at sMRU**

| Obstruction | +ve | –ve |
|---|---|---|
| Intrinsic | | |
| Stricture | 147 | 10 |
| Ureteric tumor | 6 | — |
| Extrinsic | | |
| Bladder tumor | 35 | — |
| Prostatic tumor | — | 1 |
| Retroperitoneal fibrous | 5 | — |
| Local recurrence | — | 1 |
| Ureterocele | 4 | — |

**TABLE 3**
**Comparison between Single-Shot and Standard MRU in Diagnosis of Noncalcular Urinary Obstruction**

| | No. of Renal Units | Single Shot (+ve) | Multiple Shot (+ve) | *p* Value |
|---|---|---|---|---|
| Intrinsic | 157 | 134 | 97/147 | <0.001 |
| Stricture | | 134/151 | 141/151 | |
| Ureteric tumor | | 0/6 | 6/6 | |
| Extrinsic | 46 | 9 | 44 | <0.001 |
| Bladder tumor | | 7/35 | 35/35 | |
| Prostatic tumor | | 0/1 | 0/1 | |
| Retroperitoneal fibrosis | | 0/5 | 5/5 | |
| Tumor recurrence | | 0/1 | 0/1 | |
| Ureterocele | | 2/4 | 4/4 | |
| Total | 203 | 143 | 187 | <0.001 |

## DISCUSSION

The traditional intravenous pyelography (IVP) remains the investigation of choice to reveal the detailed anatomy of the pelvicalyceal system. When IVP is contraindicated or if the kidney is of poor function, gray-scale ultrasound is useful in the diagnosis of obstruction, but its value is limited in identification of the cause[14]. However, with the advances in CT techniques, it has become the study of choice in investigation of the urinary tract.

Static-fluid T2w MRU is especially suited for imaging of the dilated urinary tract in which the large amount of water generates a good signal-to-noise ratio[10,11,12,15].

Administration of an MR contrast agent is not required in sMRU. The urographic effect is independent of the renal excretory function, so it can visualize the markedly obstructed urinary tract of a quiescent kidney[6,7,11]. The latter aspect is the most important advantage of sMRU compared with conventional IVP.

T2w MRU is an option in patients who had contrast allergy; it does not require the costly nonionic iodinated compound or prophylaxis against reaction with steroids.

Administration of intravenous contrast to diabetic patients can be avoided because these patients may have compromised renal function secondary to impaired glomerular filtration and nephrosclerosis[16].

T2w MRU is safe during pregnancy, therefore, it potentially has the ability to differentiate physiological dilatation in pregnancy from pathological dilation[17].

In the clinical practice of T2w sMRU, single-slice projection imaging with a section thickness of up to 80 mm does not have the same discernment as the multislice technique. Multislice HASTE or standard TSE sequences are more time consuming, but the acquisition of overlapping thin sections has proved to be superior for the detection of small pathological details, which can be missed on an 8-cm-thick projection MR urogram[5,8,9].

In our study, the most frequent cause of obstruction was the intrinsic stricture, representing 77% of cases, and the overall accuracy of both techniques were comparable. Therefore, we used the single-slice MRU as a localizer with different oblique that requires about 10 sec, and can detect the level and the stricture with high accuracy in most cases of ureteric obstruction. So, the study could be completed with multiple axial T2w images at the level of obstruction. In cases of intrinsic stricture due to ureteric tumor, none could be diagnosed using single-shot MRU due to its poor soft tissue detectability with the high fluid contrast that masks the soft tissue lesions. Multisection MRU could detect all cases due to high soft tissue reproducibility and resolution. For patients with suspected transitional cell carcinoma, it allows us a good opportunity to combine MRU with standard MRI in the axial plane[9], thus offering a potential alternative to CT.

Cases of ureteral stenosis seen with extrinsic compression are different from those observed in transitional cell carcinomas. A filling defect is missing. A goblet sign is not seen. The extrinsic compression of the lumen usually appears as a concentric stricture either with gradual tapering of the ureteral wall[8,11] or abrupt reduction of the ureteric caliber[15]. In our study, the single-shot MRU had a very poor sensitivity in detection of extraureteric causes of ureteric obstruction; it could diagnose only two out of four cases of a ureterocele, the two missed lesions were small and masked by the high fluid signal intensity. The multisection MRU was of high sensitivity and specificity in diagnosing extraureteric obstruction, and all cases of bladder cancer, ureterocele, and retroperitoneal fibrosis were diagnosed accurately, although one case of prostatic cancer and one case of local pelvic tumor recurrence were missed due to the small size of the lesions, i.e., they were not interpreted accurately at the MRU slice with 5-mm sections, so the relation of lesion to ureter could not be detected.

## CONCLUSION

In conclusion, T2w sMRU is a very useful technique in diagnosing noncalcular ureteric obstruction. Multisection MRU has a high diagnostic accuracy and reliability over that of the single-shot technique. The single-section technique is very rapid and useful in diagnosing ureteric strictures, so it could be used as a localizer, while multisection MRU with postprocessing MIP images is mandatory, especially in cases of suspected ureteric tumors or extraureteric causes.
